# Rutherford County Medical Society

**Published:** 1845-01

**Authors:** 


### RUTHERFORD COUNTY MEDICAL SOCIETY.

The physicians of Rutherford county, in Tennessee, have united in the organization of a society, for the advancement of medical science and the promotion of friendly professional intercourse, and are holding meetings, at which papers on the medical topography of their county are read, and all medical matters interesting to the members, are brought up and discussed. Improvement to the members, and benefit to the community, must result from this association. We cannot conceive of any method so well calculated to advance all the great objects in which the profession have a deep concern, as the formation of such societies wherever a number of physicians can be brought together. Mutual instruction, a heightened taste for study, and harmony of professional intercourse, are the certain fruits of association under these circumstances, where it is kept up and cherished in the proper spirit. We should be glad to see a well organized, well conducted Medical Society in every county of every State, in which the medical profession make so respectable a portion of the population as they do in Rutherford. We wish the Medical Society of Rutherford, and every member thereof, abundant prosperity.

